# Transcriptome analysis of genetic mechanism of growth curve inflection point using a pig model

**DOI:** 10.1016/j.gdata.2015.08.024

**Published:** 2015-09-02

**Authors:** Linyuan Shen, Shunhua Zhang, Li Zhu

**Affiliations:** College of Animal Science and Technology, Sichuan Agricultural University, Chengdu, Sichuan, China

## Abstract

Animal growth curves play an important role for animal breeders to optimize feeding and management strategies (De Lange et al., 2001 [1]; Brossard et al., 2009 [2]; Strathe et al., 2010 [3]). However, the genetic mechanism of the phenotypic difference between the inflection point and noninflection points of the growth curve remains unclear. Here, we report the differentially expressed gene pattern in pig *longissimus dorsi* among three typical time points of the growth curve, inflection point (IP), before inflection point (BIP) and after inflection point (AIP). The whole genome RNA-seq data was deposited at GenBank under the accession number PRJNA2284587. The RNA-seq libraries generated 117 million reads of 5.89 gigabases in length. Totals of 21,331, 20,996 and 20,139 expressed transcripts were identified in IP, UIP and AIP, respectively. Furthermore, we identified 757 differentially expressed genes (DEGs) between IP and UIP, and 271 DEGs between AIP and IP. Function enrichment analysis of DEGs found that the highly expressed genes in IP were mainly enriched in energy metabolism, global transcriptional activity and bone development intensity. This study contributes to reveal the genetic mechanism of growth curve inflection point.

**Table:** 

Specifications	
Organism/cell line/tissue	Pig *longissimus dorsi*
Sex	Female
Sequencer or array type	Illumina Hiseq^TM^ 2000
Data format	Raw data: fasta file, analyzed data: xlsx file
Experimental factors	Muscle from inflection point versus non inflection points.
Experimental features	Comparative transcriptomic analyses between muscles at inflection point and noninflection points.
Consent	N/A
Sample source location	Sichuan, China

## 1 Direct link to deposited data

The RNA-seq raw data has been uploaded in GEO database under the accession number GSE69113 (http://www.ncbi.nlm.nih.gov/geo/query/acc.cgi?acc=GSE69113).

The whole project was deposited at GenBank under the accession number PRJNA2284587 (http://www.ncbi.nlm.nih.gov/bioproject/PRJNA284587).

## 2 Experimental design, materials and methods

One Chinese native mountain-type pig breed, Liangshan pig, was taken as the animal model in the study (Shen et al., 2014 [4]). A total of 275 female Liangshan pigs were raised from birth to 250 days old to collect the growth traits (feed conversion rate, daily feed intake and average daily gain). The growth curve was fitted by three different non-linear models. The inflection point analysis of the growth curve suggested that the Liangshan pig reached the maximum growth rate at day 193.40. Therefore, we selected other two symmetric non-inflection points (143 days for BIP and 243 days for AIP) to explore the transcriptome diversity of muscle development. The *longissimus dorsi* muscle was harvested from 9 Liangshan pigs (3 pigs for each time point), and used for transcriptome analysis.

For RNA-Seq library preparation, total RNA was extracted from *longissimus dorsi* using TRIzol (Invitrogen, CA, USA) and further purified with RNeasy column (Qiagen, USA) according to the manufacturer's protocol. The total RNA was isolated poly (A) mRNA by poly-T oligo attached magnetic beads (Thermo-Fisher). Following purification, the mRNA was fragmented into small pieces using divalent cations under an elevated temperature. Then the cleaved RNA fragments were constructed into the final cDNA library in accordance with the protocol for the Illumina RNA ligation based method (Illumina, San Diego, USA). A reverse transcription followed by PCR was used to create cDNA constructs. The average insert size for the single-end libraries was 300 bp (± 50 bp). Then the single end sequencing (50 bp) was performed on an Illumina Hiseq2000 platform. For data analysis, the raw data containing adaptor sequences, reads with low quality sequences and unknown nucleotides N were filtered to obtain clean reads with 50 nt in length. Clean reads were then conducted for quality assessment (data shown in Table 1). These include the classification of total and distinct reads and show their percentage in the library, analyze saturation of the library and correlation analysis of biological replicates. All clean reads were mapped to the transcript sequence by bowtie (1.0.0); only 1 bp mismatch was allowed. For monitoring the mapping events on both strands, both the sense and complementary antisense sequences were included in the data collection (data shown in Table 2). The number of perfect clean reads corresponding to each gene was calculated and normalized to the number of Reads Per Kilobase of an exon model per Million mapped reads (RPKM). Based on the expression levels, the significant DEGs (differentially expressed genes) among different samples were identified with *p*-value ≤ 0.05 and log_2_fold-change|log 2 FC∣ ≥ 1. Raw and normalized data were accessible on public database: An enrichment analysis of DEGs found the immune system related genes were in the BIP stage. The energy metabolism rate, global transcriptional activity and bone development intensity were highly expressed in the inflection point period. Superior meat quality was developed in the AIP stage. The raw and normalized data of our study were accessible on public database: GEO submission number GSE69113.

## Conflicts of interest

The authors declare no conflicts of interest.

## Figures and Tables

**Table 1:** Overview of sequencing data (total reads). BIP: before inflection point; IP: under inflection point; AIP: after inflection point. CopyNum: copy number of reads.

Items	BIP-1	BIP-2	BIP-3	IP-1	IP-2	IP-3	AIP-1	AIP-2	AIP-3
Raw data	13339799	10330457	17695127	11864291	12318028	13499142	13119946	12619481	12905984
After adaptor cut	13275704	10285839	17684488	11856682	12311919	13456169	13105441	12614571	12900101
After junk filter	13244525	10263834	17662944	11838415	12297883	13430696	13085474	12601514	12886097
Valid data	13244525	10263834	17662944	11838415	12297883	13430696	13085474	12601514	12886097
CopyNum 1	13244525	10263834	17662944	11838415	12297883	13430696	13085474	12601514	12886097
CopyNum ≥ 5	7460500	5454381	10942294	6570150	6978726	7925320	8018356	7371137	7704734
CopyNum ≥ 10	6562426	4736476	9820014	5730831	6008702	6946867	7129213	6504642	6847125
CopyNum ≥ 20	5749527	4094905	8738767	4962857	5089506	6008240	6347582	5696676	6055181
CopyNum ≥ 50	4646376	3149458	7274146	3871935	3862539	4702611	5229975	4552111	4894318
CopyNum ≥ 100	3535075	2230578	5927035	2875443	2864061	3641747	4091514	3569003	3817496

**Table 2:** Overview of mapped reference gene on sequencing valid data. BIP: before inflection point; IP: under inflection point; AIP: after inflection point.

Items	BIP-1	BIP-2	BIP-3	IP-1	IP-2	IP-3	AIP-1	AIP-2	AIP-3
Mapped gene	20690	19907	20904	19907	19823	19599	19514	19263	19599
Match (unique sense) ≤ 1 mismatch									
1 gene → mapped by 1 unique sequence	1262	1342	1160	1414	1338	1376	1389	1362	1361
1 gene → mapped by n unique sequence	16256	15888	16689	15552	15827	15382	15460	15238	15535
Match (unique antisense) ≤ 1 mismatch									
1 gene → mapped by 1 unique sequence	114	106	107	164	114	104	110	106	126
1 gene → mapped by n unique sequence	105	136	132	118	108	132	105	143	134
Match (unique sense and antisense) ≤ 1 mismatch									
1 gene → mapped by n unique sequence	2953	2435	2816	2659	2436	2605	2450	2414	2443
